# IntEgrating Smoking Cessation treatment As part of usual Psychological care for dEpression and anxiety (ESCAPE): protocol for a randomised and controlled, multicentre, acceptability, feasibility and implementation trial

**DOI:** 10.1186/s40814-018-0385-2

**Published:** 2019-01-22

**Authors:** Gemma Taylor, Paul Aveyard, Kate Bartlem, Alison Shaw, Jeremy Player, Chris Metcalfe, David Kessler, Marcus Munafò

**Affiliations:** 1 Addiction and Mental Health Group (AIM), Department of Psychology, University of Bath, 10 West, Bath, BA2 7AY UK; 2 Nuffield Department of Primary Care Health Sciences, UK Centre for Tobacco and Alcohol Studies, University of Oxford, Radcliffe Primary Care Building, Radcliffe Observatory Quarter Woodstock Road, Oxford, OX2 6GG UK; 3 School of Psychology, University of Newcastle, Behavioural Sciences Building, University Drive, Callaghan, 2308 Australia; 4 Population Health, Hunter New England Local Health District, Wallsend Health Services, Booth Building, Longworth Avenue, Wallsend, NSW 2287 Australia; 5 Centre for Academic Primary Care, Bristol Medical School, Department of Population Health Sciences, Canynge Hall, University of Bristol, 39 Whatley Road, Bristol, BS8 2PS UK; 6 Jeremy Player, Solutions 4 Health, 7200 The Quorum, Oxford Business Park, Garsington Road, Oxford, OX4 2JZ UK; 7 Bristol Randomised Trials Collaboration, Population Health Sciences, Bristol Medical School, Canynge Hall, 39 Whatley Road, Bristol, BS8 2PS UK; 8 Centre for Academic Primary Care, Bristol Medical School, Department of Population Health Sciences, University of Bristol, Oakfield House, Oakfield Grove, Bristol, BS8 2BN UK; 9 UK Centre for Tobacco and Alcohol Studies, School of Experimental Psychology, University of Bristol, 12a Priory Road, Bristol, BS8 1TU UK; 10 MRC Integrative Epidemiology Unit, Oakfield House, Oakfield Grove, Bristol, BS8 2BN UK

## Abstract

**Background:**

People with depression/anxiety are twice as likely to smoke and are less responsive to standard tobacco treatments, leading to a reduced life expectancy of up to  13.6 years compared to people without depression/anxiety. However, this group of smokers is motivated to quit, and as a result of quitting smoking, their depression/anxiety is likely to improve. In England, people with depression/anxiety are referred to a primary care-based psychological therapies service known as ‘Improving Access to Psychological Therapies’ (IAPT), which could offer smoking cessation treatment as part of usual care but currently does not. In this study, we aim (1) to establish the feasibility and acceptability of delivering a smoking cessation treatment alongside IAPT usual care and (2) to establish the feasibility of a multi-centre randomised trial to compare the combined smoking cessation and IAPT treatment to usual IAPT treatment alone.

**Methods:**

A randomised and controlled, multi-centre trial to test the acceptability, feasibility and implementation of smoking cessation treatment as offered alongside usual IAPT care, compared to usual care alone, with nested qualitative methods. We will include adult daily smokers with depression/anxiety, who would like help to quit smoking and are about to start IAPT treatment. Follow-up will be conducted at 3-months after baseline. The main outcome will be retention in the smoking cessation treatment. Secondary outcomes are smoking-related (biochemically-verified 7-day point prevalence smoking cessation, number of cigarettes smoked per day, Heaviness of Smoking Index), mental health-related (PHQ-9), service-related (number of ‘Did Not Attends’, number of planned and completed IAPT sessions), acceptability and feasibility (participant and clinician acceptability and satisfaction of intervention as assessed by questionnaires and qualitative interviews, interviews will also explore acceptability and feasibility of data collection procedures and impact of smoking cessation treatment on usual care and mental health recovery) and implementation-related (intervention delivery checklist, qualitative analysis of intervention delivery).

**Discussion:**

If the intervention is shown to be acceptable, feasible and suitably implemented, we can conduct a randomised controlled trial. In a future trial, we would examine whether adding smoking cessation treatment increases smoking abstinence and improves depression and anxiety more than usual care, which would lead to long-term health improvement.

**Trial registration:**

ISRCTN99531779

**Electronic supplementary material:**

The online version of this article (10.1186/s40814-018-0385-2) contains supplementary material, which is available to authorized users.

## Background

Smoking is the world’s leading cause of preventable illness and death [1]. One in every two smokers will die of a smoking-related disease, unless they quit [2, 3]. In high-income countries, smoking prevalence has decreased from 46% during the 1970s to about 19% in recent years [4–6]. However, smoking prevalence amongst people with mental disorders has declined only slightly and is currently around 32.0% [7, 8]. In the UK, recent estimates indicate that 33.7% of people with depression, and 28.9% of people with anxiety smoke [7, 8]. They are more heavily addicted, suffer from worse withdrawal [9, 10] and experience a 19% reduction in the odds of quitting (odds ratio 0.81, 95% CI 0.67 to 0.97) [10], even though they are motivated to quit [11]. These inequalities contribute to a reduction in life expectancy of up to 13.6 years for people with depression/anxiety compared to the general population [12, 13].

Traditionally, mental health and addictive behaviours have been treated separately. More recently, there has been a movement towards interventions that target both mental health and addiction [14–16]. Studies in patients with severe mental disorders or alcoholism have shown that treating addiction and mental health in parallel results in meaningful improvements in both [17–21]. van der Meer and colleagues conducted a Cochrane review of smoking cessation treatments for people with current and historical depression [21] and found that adding psychosocial mood management to usual smoking cessation treatment (e.g., nicotine replacement therapy) moderately increased cessation rates when compared to usual smoking treatment alone (risk ratio of 1.47; 95% confidence interval 1.13 to 1.92) [21], thus highlighting the importance of psychological support during a quit attempt for this group.

New evidence suggests that smoking can cause mental illness [45], and that stopping smoking is associated with mental health benefits equal to anti-depressant treatment [22]. In England, people with depression/anxiety have access to psychological therapy services, known as ‘Improving Access to Psychological Therapies’ (IAPT), in which service users receive psychological therapies for depression and anxiety. IAPT may be an ideal platform for delivering smoking cessation treatment. However, there have been no trials comparing the effectiveness of offering smoking cessation treatment alongside usual care, and therefore, it is unknown if such a treatment is feasible and acceptable to service users and therapists, and can be implemented into the service. We have spent the last 9 months designing an intervention with stakeholders to enhance intervention suitability. In this study, we aim to assess: (1) the feasibility of recruiting and retaining participants, collecting data required for a full-sized RCT, and randomisation procedures; (2) the acceptability of data collection procedures and the smoking cessation treatment as delivered alongside usual IAPT care, as perceived by IAPT therapists and study participants; and (3) implementation of the smoking cessation treatment programme.

## Methods

### Design

A randomised and controlled multicentre feasibility trial with nested qualitative research will be conducted to test the acceptability, feasibility and implementation of smoking cessation treatment offered alongside usual psychological care. The study has been registered on the World Health Organization's International Clinical Trials Registry Platform (ID: ISRCTN99531779). Any changes to the methods outlined in this protocol will be outlined in the report of the study findings.

### Ethical review

This study received ethics approval from the National Health Service (NHS) Research Ethics Committee on 19/03/2018 (IRAS ID: 239339). In the case that there are any protocol modifications, these will be submitted for ethical review.

### Setting

This is a multicentre study involving two Improving Access to Psychological Therapies (IAPT) sites in the UK (i.e., Bristol and Oxford). IAPT is a NHS primary-care based psychological therapy service (https://www.england.nhs.uk/mental-health/adults/iapt/). IAPT offers low- and high-intensity evidence-based therapies (e.g., cognitive behavioural therapy (CBT)) to people with common mental disorders. Psychological treatment is delivered by therapists named ‘psychological wellbeing practitioners’ (PWPs) and usually consists of six 30–45-minute sessions, delivered over the telephone or face-to-face (further details below).

### Project timeline

**Fig. 1:**
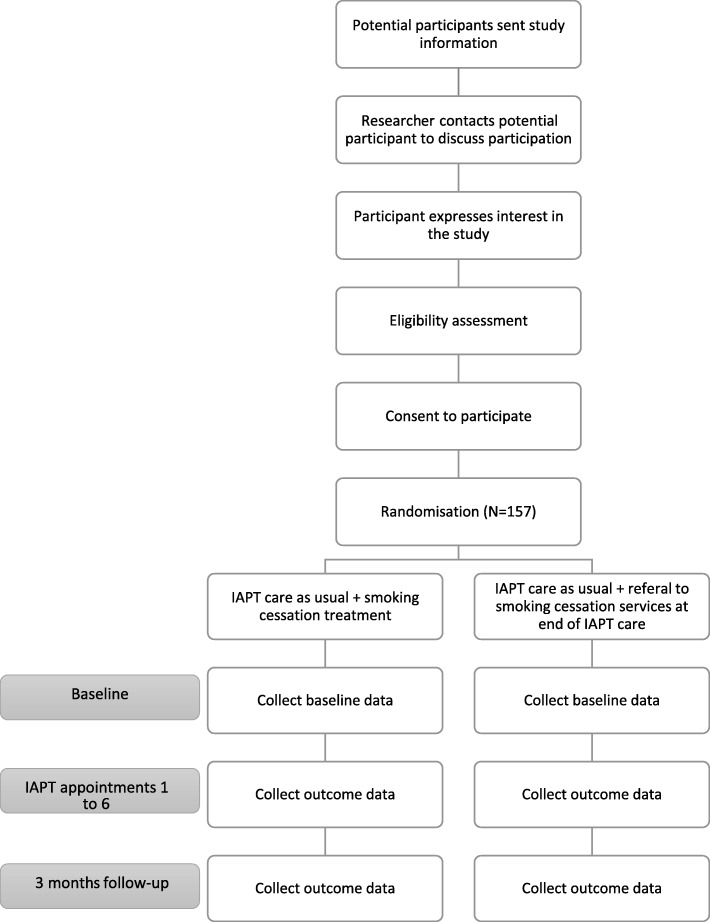
Flow chart of study events

The study will be undertaken over 18 months, and the final follow-up will be in September 2019 (see Additional file 1 for a detailed study timeline and Table 1 and Fig. 1 for a ‘Standard Protocol Items: Recommendations for Interventional Trials (SPIRIT)’ [23] schedule of enrolment, interventions and assessments, and study flow chart).

**Table 1:**
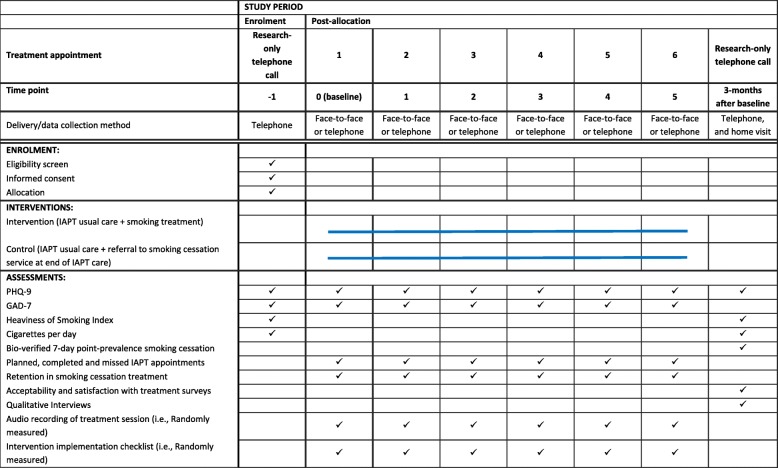
SPIRIT schedule of enrolment, interventions and assessments

### Eligibility criteria

#### Participants (IAPT service users)

The inclusion criteria are as follows:

- Aged 18 years or older
- Has current diagnosis of depression (clinician-administered PHQ-9 score of ≥ 10) and/or anxiety (clinician-administered GAD-7 score of ≥ 8) (note: other mental health comorbidities are allowable)
- Self-reported, daily tobacco smoker of at least 1 year
- Interested in receiving help to quit smoking tobacco
- Eligible for IAPT treatment on a one-to-one basis over the telephone or face-to-face
- About to start psychological therapy for depression/anxiety in IAPT

The exclusion criteria are as follows:

- Already started IAPT treatment
- Considered too unwell by the research or IAPT team
- Pregnant or breastfeeding

#### Psychological wellbeing practitioners (PWPs, IAPT therapists)

The inclusion criteria are as follows:

- Aged 18 years or older
- Have provided psychological treatment to people with depression/anxiety in IAPT for at least 2 years
- Available to attend training in delivering the smoking cessation intervention
- Non- or ex-smoker

### Withdrawal of participants

Patient and PWP participants can withdraw from the study at any time, and they can withdraw their data from the study at any time during the study period.

### Payments to participants

Participants will not be paid for their contribution to the study.

### Intervention arm

In both the intervention and control groups, participants will receive an evidence-based psychological therapy (i.e., CBT), typically lasting 30–45 minutes per session, delivered over 6 sessions. In the intervention group only, participants will receive a multi-component smoking cessation treatment that includes behavioural, psychological and pharmacological support. PWPs will deliver the smoking cessation treatment as parallel to usual IAPT care (see Table 2). The smoking cessation treatment package is an adapted version of the National Centre for Smoking Cessation and Training’s (NCSCT) standard treatment programme [24]. The NCSCT standard treatment programme is based on the most up-to-date evidence available, is supported by the National Institute for Clinical Excellence and is proven to be cost-effective in NHS settings [25, 26]. The smoking cessation treatment will be delivered over the telephone or face-to-face (i.e., depending on usual care) and will be delivered on an individual basis. Based on interviews and consultation with stakeholders, the intervention has been altered to best fit IAPT usual care, and service user needs, while being mindful of the NCSCT’s standard treatment programme.

**Table 2:** Overview of treatment components

Session	1	2	3–5	6
Duration in minutes	10–15	10–15	5–10	5–10
Smoking cessation treatment session	Pre-quit	Quit day	Follow-up	Final
Address participant beliefs about smoking and mental health	✓	✓	✓	✓
Inform the participant about the treatment programme	✓			
Assess current smoking	✓			
Assess past quit attempts	✓			
Explain how smoking dependence develops and assess nicotine dependence	✓			
Explain the importance of abrupt cessation and the ‘not a puff’ rule	✓	✓	✓	✓
Inform the participant about withdrawal symptoms	✓			
Discuss stop smoking medications/products	✓			
Set the quit date	✓			
Prompt a commitment from the participant	✓	✓		
Check on participant progress			✓	✓
Confirm participant readiness and ability to quit		✓		
Confirm that the participant has a sufficient supply of stop smoking medication/products		✓	✓	✓
Give participant NRT vouchers or refer to pharmacy/GP for varenicline	✓	✓	✓	✓
Enquire about medication use			✓	✓
Discuss withdrawal symptoms and cravings, and how to cope		✓	✓	
Advise on changing routine		✓		
CO-monitoring (i.e., site dependent)	✓	✓	✓	✓
Discuss how to address the issue of the participant's smoking contacts and how the participant can get support during their quit attempt		✓		
Discuss any difficult situations experienced and methods of coping			✓	✓
Address any potential high-risk situations in the coming week		✓	✓	
Discuss plans and provide a summary	✓	✓	✓	✓

#### Duration and number of sessions

Participants will receive 5–15 minutes of smoking cessation treatment per IAPT appointment, delivered over 6 IAPT usual care appointments. In Bristol, PWPs will deliver smoking cessation treatment within the usual care timeframe. In Oxford, PWPs will be allocated an extra 5–15 minutes per IAPT appointment to deliver the smoking cessation treatment. We predict that treatment will last approximately 12 weeks; however, this is subject to the progress of the participant's IAPT care. During the first two IAPT appointments, 10–15 minutes will be allocated to delivering the smoking cessation treatment, and the remaining time will be used to deliver IAPT usual care. For the following four IAPT appointments, 5–10 minutes will be used to deliver the smoking cessation treatment, and the remaining time will be used to deliver IAPT usual care.

#### Behavioural and psychological components

PWPs will offer behavioural support as outlined in the NCSCT's standard treatment programme [24]. See Table 2 for an outline of the modified version of the standard treatment programme that will be delivered in this study. In addition to behavioural support, participants will receive psychoeducation about smoking and mental health. New evidence suggests that smoking can cause mental illness [45], and that stopping smoking is associated with mental health benefits equal to anti-depressant treatment [22]. This can partially be explained by breaking the smoking withdrawal cycle—in sum, nicotine has a short half-life, and smokers start to experience the psychological symptoms of withdrawal soon after having a cigarette, for example, symptoms of low mood and anxiety. Therefore, the smoker is in a constant state of withdrawal, with short periods of relief only when they are actively smoking and shortly after finishing a cigarette. It may be that smokers mistake the ability of smoking to relieve withdrawal symptoms to its ability to relieve stress, anxiety and low mood. [27, 28]. Participants will receive an information pack about quitting smoking and mental health via email, leaflet or post.

#### Nicotine replacement therapy (NRT)

NRT will be provided at cost of prescription, or free-of-charge (i.e., depending on site), alongside behavioural support to use NRT (NCSCT and the British National Formulary [24, 29]). NRT will be posted or participants will be given a prescription request to present at their local pharmacy (i.e., depending if IAPT care is delivered over the telephone or face-to-face). In both sites, participants will receive an information pack that includes text and video instructions on how to take the NRT. Participants who self-report having diabetes mellitus, renal impairment or hepatic impairment will be referred to their GP or pharmacy prescriber for NRT [29].

Participants will be provided with two NRT products, and the participant will have the opportunity to alter the dose or type of NRT [24]. As per the NCSCT’s standard treatment programme, participants will be provided with nicotine patches, and the strength will depend on the level of nicotine dependency as assessed using the Heaviness of Smoking Index [30]. A second NRT product will be recommended to help combat cravings—participants will have a choice of a lozenge, gum, microtab, nasal spray, mouth-spray, mini lozenges, or strips, with strength depending on the level of nicotine dependency. See Additional file 1 for the list of NRT products available.

#### Varenicline

If the participant prefers not to use NRT or has previously used NRT as part of a behavioural treatment and failed to quit, they will have the option of using varenicline, pending confirmation from their GP/pharmacy. The participant’s GP or pharmacist prescriber will prescribe varenicline at their discretion. Participants will receive behavioural support for use of varenicline as outlined by the NCSCT [24] and in Table 2.

#### E-cigarettes

PWPs will provide behavioural support to participants who choose to use e-cigarettes to aid their quit attempt. This follows recommendations from Public Health England [31]. However, the study will not fund the purchase of e-cigarettes or accessories.

#### Differences between NCSCT’s standard treatment programme for smoking cessation and the smoking cessation treatment offered in this study

To tailor the smoking cessation treatment to IAPT usual care, we have interviewed PWPs, service managers and service users (NHS National Research Ethics Service review ID:225399. i.e., under write-up) and consulted with current and prospective commissioners for IAPT services. Based on interviews and consultations, we modified the NCSCT’s standard treatment programme as follows:

1. Shortened total treatment time from 110 minutes to 40 to 70 minutes to meet IAPT service requirements
2. CO-monitoring will not be part of the intervention in one of the sites, as IAPT care is predominately telephone-based in one of the two sites
3. There will be an emphasis on addressing the relationships between smoking, the withdrawal cycle and links to mental health

#### Integrating smoking cessation treatment into IAPT usual care

IAPT is predominately based on a guided self-help model. The main aim of guided self-help is to support the service user in learning how to make positive changes to their behaviour and thinking, to help improve their mental health. With support from their PWP, service users learn a toolkit of coping strategies to help them to become their own therapist, so that they can manage their mental health effectively and prevent future setbacks. Pre-trial interviews with PWPs and IAPT service users, and service managers have identified that there are many psychological theories and techniques that PWPs regularly use that provide opportunities to deliver behavioural and psychological support to aid smoking cessation, for example, motivational interviewing, psychoeducation and behavioural activation.

### Control arm

Participants in the control arm will receive IAPT usual care, plus contact details for their local smoking cessation service during their final IAPT appointment.

### PWP training programme

A maximum of ten PWPs per site will be trained to deliver the treatment. PWPs will take part in the following training:

1. NCSCT level 1 online training and assessment programme
2. NCSCT level 2 face-to-face training
3. An overview about the importance of randomised trial methodology and study procedures (e.g., data recording)
4. A researcher will visit each service once a month to listen in sessions and offer feedback to PWPs

### Outcomes

The trial aims to assess: (1) the feasibility of recruiting and retaining participants, collecting data required for a full-sized RCT, and randomisation procedures, (2) the acceptability of data collection procedures and the smoking cessation treatment as delivered alongside usual IAPT care, as perceived by PWPs and study participants, and (3) implementation of the smoking cessation treatment programme.

#### Main acceptability and feasibility outcome

- *Study completers*: Participants will be considered a completer if they continue with smoking cessation treatment up until the point of smoking cessation, a quit attempt, or completion of IAPT care. The proportion of study completers will be calculated by:

$$ \frac{N\ \mathrm{study}\ \mathrm{completers}}{N\ \mathrm{randomised}\ \mathrm{at}\ \mathrm{baseline}} $$

#### Secondary acceptability and feasibility outcomes

- Recruitment into the trial
- Participant and PWP acceptability and satisfaction of smoking cessation treatment (Modified version of the Stop Smoking Service Client Satisfaction Survey [32] and Modified version of a clinician self-report intervention acceptability questionnaire [33])
- Qualitative interviews with PWPs will explore PWP perceptions of:
  - Acceptability of the smoking cessation treatment
  - Acceptability of data collection procedures
  - Positive and negative impacts of smoking cessation treatment on IAPT usual care and mental health recovery
- Qualitative interviews with participants will explore participant perceptions of:
  - PWP ability to deliver the smoking cessation treatment
  - Acceptability of the smoking cessation treatment
  - Positive and negative impacts of smoking cessation treatment on IAPT usual care and mental health recovery

#### Implementation outcomes

- *Intervention Implementation Checklist*: We will listen to a proportion of smoking cessation treatment sessions and note which intervention components were delivered or not (Table 2)
- Observations and listening to intervention delivery recordings

#### We will check data completeness for the following outcomes

Smoking-related:

- Carbon monoxide (CO)-verified 7-day point prevalence smoking cessation (i.e., validated by exhaled carbon monoxide concentration of < 10 ppm (ppm) [34]. CO-testing will be available on both sites via a 3 month follow-up home visit.
- Number of cigarettes per day (CPD)
- Heaviness of Smoking Index (HSI) [35]

Mental health-related:

- Patient Health Questionnaire (PHQ-9) [36] score

Service-related:

- Number of ‘Did Not Attends’
- Number of planned, completed and missed IAPT appointments

### Sample size

#### Trial

Assuming a sample size of 100 participants at follow-up, we used a standard formula to calculate the binomial exact confidence interval [37, 38]. The study will have sufficient power to estimate that 40% or more participants will continue with smoking cessation treatment in the intervention arm with a 95% confidence interval of 26% to 55% (i.e., assuming the true rate is 40%).

Therefore, assuming that about 36% of participants will be lost to follow-up [39], this study will require a sample size of 157 at baseline:

$$ \left(\mathrm{i}.\mathrm{e}.,157-\left(157\ \left(\frac{36}{100}\right)\right)=100\right)\Big) $$

#### Interviews

We will aim to interview all PWPs who deliver the smoking cessation treatment and 20 trial participants, or until saturation is reached [40]. We will use a purposeful sampling method and aim for variation across the sites and participant final smoking status.

### Recruitment and consent procedures

#### Trial

Researchers will search for potentially eligible participants via patient management software. Potentially eligible participants will be posted study information at least 2 weeks prior to their first IAPT appointment and contacted by a researcher before their first IAPT appointment to discuss eligibility and gain oral informed consent. The trial consent form is available in the Additional file 1.

#### Audio recording of treatment sessions

Sessions are recorded as part of IAPT usual care, and written informed consent is gained on the same day as treatment by PWPs.

#### Interviews

During the 3 month follow-up, trial participants will be recruited into interviews. PWPs involved in intervention delivery will be recruited into interviews after they have finished delivering the smoking cessation intervention to their final participant. Written or oral informed consent will be obtained on the day of the interview.

### Randomisation and intervention assignment methods

#### Sequence generation

The randomisation sequence will be generated by a statistician using Stata software. Randomisation will be stratified by site and blocked, and participants will be randomised using a 1:1 algorithm to ensure an equal number of participants in the treatment and control arms.

#### Allocation concealment mechanism

Allocation concealment will be ensured as the randomisation code will not be released until the IAPT service user has been recruited into the trial, which takes place after participant eligibility has been assessed, participant identifier has been recorded, and consent gained to take part in the trial and to being randomly allocated to treatment condition.

#### Implementation

Randomisation will be requested via RedCap by the researcher who recruited and consented the participant into the trial. RedCap will send a response to the researcher informing them which treatment the participant will be receiving. Randomisation can only be requested once and after participant identifier, eligibility and consent have been recorded, and therefore, implementation cannot be influenced by the PWP, participant, the research or clinical team.

### Blinding

Due to the nature of the intervention, it is not possible to blind participants and PWPs who deliver the intervention. Quantitative 3 month follow-up outcome assessments will be conducted by a researcher who will be blinded to treatment allocation. Qualitative interviews will be conducted after quantitative outcomes assessment, as it is possible that the researcher will become unblinded during interviews. Outcome assessor blinding will also be verified by self-report at final follow-up.

### Data collection

See Additional file 1 for full data management plan.

#### Quantitative data collection

Table 1 and Fig. 1 present a schedule of data collection. Most participant data will be collected as part of usual care. Baseline and 3 month follow-up data will be collected by a researcher. Usual care data will be recorded by PWPs using patient management software and then extracted and input into RedCap by researchers (Table 3).

**Table 3:** List of variables, and who the variables will be collected by

Category	Variable	Collected by who*	Are data collected as part of routine care?
Eligibility	Eligibility criteria met	Researcher	No
Demographics	Date of birth	PWP/researcher	Yes
	Ethnicity	PWP/researcher	Yes
	Gender	PWP/researcher	Yes
	Education	PWP/researcher	Yes
Mental health	Comorbid mental health conditions	PWP/researcher	Yes
	PHQ-9	PWP/researcher	Yes
	GAD-7	PWP/researcher	Yes
Smoking	Smoking status	PWP/researcher	Yes, but not regularly
	Smoking history	PWP/researcher	No
	Cigarettes per day	PWP/researcher	No
	Bio-verification of smoking status	Researcher	No
	Smoking cessation medication used in intervention	PWP	No
Service	Participant drop-out of IAPT	PWP	Yes
	Planned, completed and missed IAPT appointments	PWP/researcher	Yes
Acceptability and feasibility	Retention in smoking cessation intervention	PWP	No
	Participant satisfaction with smoking cessation intervention	PWP/researcher	No
	Clinician satisfaction with smoking cessation intervention	Researcher	No
	Qualitative Interviews	Researcher	No
Implementation	Audio recording of treatment session	PWP	Yes
	Intervention implementation checklist	Researcher	No

*PWP = Psychological wellbeing practitioner

#### Qualitative data collection

We will collect two types of qualitative data:

1. Audio recordings of intervention delivery
2. Post-intervention interviews with PWPs and participants

Intervention delivery audio recordings will be taken as part of usual care, and only relevant sections, as decided by a researcher, will be used for analysis (i.e., where smoking cessation treatment is being delivered). We will have access to these recordings and will sample 30 random sessions to assess implementation.

Interviews will be conducted by a researcher, ideally in-person, but also over the telephone upon request. Interviews will last no longer than 60 minutes and will be conducted using flexible topic guides to ensure that the same broad topics are covered in all interviews, while allowing flexibility for interviewees to introduce new issues [41]. Topic guides will be modified as necessary throughout the course of the interviews to reflect findings as they emerge. The interviewer will use open-ended questioning techniques to elicit participants’ own experiences and views, and participants will be asked to provide examples to avoid reliance on ‘hypothetical’ accounts. Interviews will be anonymised to protect confidentiality. All interview data will be transcribed using a university-approved service.

### Post-trial care

If the participant would like to use smoking cessation medication/products beyond their IAPT treatment, they will be able to obtain a prescription from their GP or local smoking cessation service. Participants will be required to pay a prescription dispensing fee, unless they are exempt from paying for their prescriptions through the NHS. If the participant was not successful in their quit attempt, the PWP will provide them with contact details to a local stop smoking service.

### Adverse events

PWPs will check in with participants about the progress of their quit attempt and the stop smoking medication/products that they are using. In the case that a participant is experiencing any unusual symptoms or events, PWPs will use the NHS ‘Decision Tree for Adverse Event Reporting’ (see Additional file 1) to class and action adverse events (i.e., any unfavourable and unintended signs, symptoms or a disease associated with treatment). Adverse event(s) will be recorded in the study file with a note that will identify when the event occurred, the details of the adverse event(s), any potential study relation, action taken and resolution/closure of the adverse event(s). An assessment of seriousness will be made and will be reported to the Chief Investigator and a Research Governance Officer.

### Patient and public involvement

During study conceptualisation, the research aims and study design were peer-reviewed by several members of the public, and were presented to the UK Centre for Tobacco and Alcohol Studies Smokers’ Panel and the Elizabeth Blackwell Institute’s Public Advisory Group for feedback. In general, the study’s concept was well received, understood, and thought to be an important area of research. We will invite a patient member to trial management meetings and involve patient and public panels in analysis and dissemination.

### Participant benefit

By taking part in this research, participants will assist in the design and planning of an intervention to help others quit smoking. Also, by choosing to take part, participants will increase their chances of quitting smoking, which is the single most important change one can make to improve their health.

### Quantitative analysis

Analyses will be conducted using Stata software.

#### Primary and secondary feasibility measures

We will present the number of participants who were screened, randomised, treated, and followed up for primary outcome in a CONSORT-style flow chart. The proportion of study completers, by trial arm, will be calculated as:

$$ \frac{N\ \mathrm{study}\ \mathrm{completers}}{N\ \mathrm{randomised}\ \mathrm{at}\ \mathrm{baseline}} $$

#### Piloting main trial outcomes

The outcome measures which we intend to include in the main trial will be piloted in this feasibility study. We will present summary statistics by trial arm for each measure at baseline and 3 month follow-up. Estimated intervention effects will be presented with 95% confidence intervals, these estimates being calculated using regression models that include study site, the patient’s PWP and baseline GAD-7 and PHQ-9 scores. Linear regression will estimate differences in means for continuous measures, and logistic regression will estimate odds ratios for binary measures. As this is a feasibility study, these estimates will be too imprecise to support definitive conclusions, but will be inspected for evidence of a benefit of combining the smoking cessation treatment programme  with IAPT usual care.

#### Sensitivity analyses

(1) We will conduct home visits with all participants to CO-verify self-reported quits; however, it is possible that some participants may decline/be unavailable for a home-visit and therefore, in those cases, we will not be able to obtain biological data. To account for this, we will compare estimates derived from CO-verified and self-reported 7-day point prevalence smoking cessation. (2) Where data permit, we will compare the approaches in Bristol and Oxford for their acceptability and feasibility.

We will develop a full statistical plan ahead of the data analysis.

### Qualitative analysis

We will conduct two qualitative analyses: (1) analysis of intervention delivery recordings and (2) analysis of qualitative interviews with PWPs and study participants.

Qualitative data will be analysed using a thematic approach, following guidance outlined by Braun and Clarke [41]; thematic analysis will allow for both anticipated themes (i.e., deductive coding) and emergent themes (i.e., inductive coding). All data will be anonymised, and to ensure the quality of data transcription, a researcher will do a 50% check of audio data against the transcripts. NVivo software will be used to code and apply the analytical framework.

#### Coding

One researcher will read the transcripts in full and will re-listen to the audio recordings before coding the transcripts, and then two researchers will read and conduct a preliminary coding of a subset (i.e., a paraphrase, label). Codes can refer to substantive things (e.g., behaviours, incidents, structures), values (e.g., beliefs about smoking, treatment, rights), emotions (e.g., sadness, happiness) and more methodological elements (e.g., interviewee found something difficult to explain or became emotional) [42].

#### Developing a framework

After coding three transcripts, researchers involved in coding will meet to compare the labels that they have applied and agree on a set of codes to apply to all remaining transcripts. Codes will be grouped together into categories, which are then clearly defined. The first framework will act as a working analytical framework as this may change after further coding of new transcripts. After initial coding of all interviews using the working analytical framework, all interviews will be re-read, and codes re-examined for overlap and distinctiveness, and a final framework of themes agreed. This framework will form the basis for the structuring and presentation of themes when writing up the qualitative findings.

#### Applying the analytical framework

The framework will be applied by indexing interview transcripts using the existing categories and codes. Each code will be assigned an abbreviation and will be given a description, to ensure consistent application of codes across the dataset. We will adopt a flexible approach, and some codes may be grouped/merged or split as additional transcripts are coded.

## Discussion

In this protocol, we describe a feasibility study that will examine both the feasibility of the intervention and the feasibility of comparing the intervention with IAPT usual care in a full randomised controlled trial (RCT).

### Strengths and limitations

In our previous study, we interviewed PWPs, service managers and service users (NHS National Research Ethics Service review ID: 225399, i.e., under write-up), and consulted with current and prospective commissioners for IAPT services in the aim of tailoring the smoking cessation treatment to IAPT usual care. The smoking cessation treatment package used in this study is an adapted version of the National Centre for Smoking Cessation and Training’s (NCSCT) standard treatment programme [24]. The NCSCT standard treatment programme is based on the most up-to-date evidence available and is proven to be cost-effective in NHS settings [25]. We tailored the NCSCT treatment package to best suit the needs of the target population and IAPT service. To optimise the likelihood of successful delievery of the smoking cessation intervention in these settings, we altered the NCSCT standard treatment programme in three ways:

1. We shortened the total treatment time from 110 minutes to 40 to 70 minutes to meet IAPT service requirements. It is possible that shortening treatment time may limit the effectiveness of the intervention. However, a recent Cochrane review found no clear evidence that decreasing the *duration* of personal contact time altered the effect of combined behavioural and pharmacological treatment for smoking cessation [43]. There is an association between *number of contacts* offered for cessation and increased chances of quitting; however, a Cochrane review found that this is not a dose-response relationship and that offering four or more contacts was more effective than offering three or fewer contacts [44]; therefore, participants in our study will be receiving an adequate number of contacts (i.e., six appointments).
2. Carbon monoxide (CO) -monitoring will not be part of the intervention in one of the sites, as IAPT care is telephone-based in one of the two sites. During co-design, we explored whether or not it was possible to use CO-monitors in the behavioural programme; however, feedback from PWPs and service managers was unanimous and indicated that it was not feasible to implement CO-monitoring these settings. We cannot predict whether or not this will alter the effectiveness of the smoking cessation intervention, and our pilot and feasibility study will not be large enough to explore the impact of this on intervention effectiveness. However, if we find that the intervention is feasible, accepted and suitably implemented, this is something that we can adjust for in a definitive RCT.
3. One aspect of this study that is not included in the NCSCT Standard Treatment Programme but will be added to the IAPT smoking cessation treatment will be psychoeducation about smoking and mental health. New evidence suggests that smoking can cause mental illness [45], and that stopping smoking is associated with mental health benefits, potentially equivalent to taking anti-depressants [22]. This evidence can partially be explained by breaking the tobacco withdrawal cycle [27, 28]. In summary, nicotine has a short half-life, and smokers start to experience the psychological symptoms of withdrawal soon after having a cigarette, for example, symptoms of low mood and anxiety. Therefore, the smoker is in a constant state of withdrawal, with short periods of relief only when they are actively smoking and shortly after finishing a cigarette. It may be that smokers mistake the ability of tobacco to relieve withdrawal symptoms to its ability to relieve stress, anxiety and low mood [27, 28]. PWPs will integrate this evidence into treatment sessions.

## Conclusion

This study is expected to lead to new insights on whether or not it is possible to deliver a collaboratively designed smoking cessation treatment programme alongside usual IAPT care as part of a holistic package of mental health treatment. This study will also help determine whether it is possible to evaluate integrating smoking cessation treatment into  IAPT usual care in a full RCT.

## Additional file

Additional file 1: Online appendix for the ESCAPE trial. (DOCX 125 kb)
