# Supplementary material for: IntEgrating Smoking Cessation treatment As part of usual Psychological care for dEpression and anxiety (ESCAPE): protocol for a randomised and controlled, multicentre, acceptability, feasibility and implementation trial
Source: Pilot Feasibility Stud. 2019 Jan 22;5:16. doi: 10.1186/s40814-018-0385-2 (PMC6343330; doi:10.1186/s40814-018-0385-2)

**ONLINE APPENDIX**

Table of Contents

[Data management plan 4](#_Toc533063738)

[Description of the data 4](#_Toc533063739)

[Format and scale of the data 4](#_Toc533063740)

[Methodologies for data collection/ generation 4](#_Toc533063741)

[Data quality and standards 6](#_Toc533063742)

[Data preservation strategy and standards: 6](#_Toc533063743)

[Data security and confidentiality of potentially disclosive information 7](#_Toc533063744)

[Formal information/data security standards: 7](#_Toc533063745)

[RedCap security: 7](#_Toc533063746)

[Main risks to data security: 7](#_Toc533063747)

[Data sharing and access 8](#_Toc533063748)

[Suitability for sharing: 8](#_Toc533063749)

[Discovery by potential users of the research data: 8](#_Toc533063750)

[Governance of access: 8](#_Toc533063751)

[The study team’s exclusive use of the data: 8](#_Toc533063752)

[Restrictions or delays to sharing, with planned actions to limit such restrictions: 8](#_Toc533063753)

[Regulation of responsibilities of users: 8](#_Toc533063754)

[Informed consent form 9](#_Toc533063755)

**List of Tables and Figures**

|  | Page |
| --- | --- |
| Figure 1 | 2 |
| Table 1 List of NRT products available to participants | 3 |
| Figure 2 Flowchart of data methods and lifecycle | 5 |
| Figure 3 Decision tree for adverse event reporting (non-CTIMPS) | 10 |

**Figure 1 Projected timeline - Study GANT chart**

|  | Apr 2018 | May 2018 | Jun 2018 | Jul 2018 | Aug 2018 | Sept 2018 | Oct 2018 | Nov 2018 | Dec 2018 | Jan 2019 | Feb 2019 | Mar 2019 | Apr 2019 | May 2019 | Jun 2019 | Jul 2019 | Aug 2019 | Sept 2019 |
| --- | --- | --- | --- | --- | --- | --- | --- | --- | --- | --- | --- | --- | --- | --- | --- | --- | --- | --- |
| Trial set-up |  |  |  |  |  |  |  |  |  |  |  |  |  |  |  |  |  |  |
| Recruitment |  |  |  |  |  |  |  |  |  |  |  |  |  |  |  |  |  |  |
| Intervention |  |  |  |  |  |  |  |  |  |  |  |  |  |  |  |  |  |  |
| Final follow-up |  |  |  |  |  |  |  |  |  |  |  |  |  |  |  |  |  |  |
| Interviews |  |  |  |  |  |  |  |  |  |  |  |  |  |  |  |  |  |  |
| Trial close |  |  |  |  |  |  |  |  |  |  |  |  |  |  |  |  |  |  |
| Analyse data |  |  |  |  |  |  |  |  |  |  |  |  |  |  |  |  |  |  |

| **Table 1 List of NRT products available to participants** | | |
| --- | --- | --- |
| **Brand** | **Nicotine Replacement Product** | **Strength** |
| NICORETTE | Cools Lozenge | 2mg, 4mg |
|  | Fruitfusion Gum | 2mg, 4mg, 6mg |
|  | Freshmint Gum | 2mg, 4mg |
|  | Original Gum | 2mg, 4mg |
|  | Icy White Gum | 2mg, 4mg |
|  | Inhalator | 15mg |
|  | Invisi Patch | 10mg |
|  | Invisi Patch | 25mg |
|  | Microtab |  |
|  | Nasal Spray | 10ml |
|  | Quickmist Mouthspray | 1mg |
| Nicotinell | Patch Step 1 | 21mg |
|  | Patch Step 2 | 14mg |
|  | Patch Step 3 | 7mg |
|  | Coated Gum Fruit | 2mg, 4mg |
|  | Coated Gum Mint | 2mg, 4mg |
|  | LOZENGE | 1mg, 2mg |
| NiQuitin | CLEAR PATCH Step One | 21mg |
|  | CLEAR PATCH Step Two | 14mg |
|  | CLEAR PATCH Step Three | 7mg |
|  | CLEAR PATCH Step One | 21mg |
|  | CLEAR PATCH Pre-Quit | 21mg |
|  | CLASSIC PATCH Step One | 21mg |
|  | CLASSIC PATCH Step Two | 14mg |
|  | CLASSIC PATCH Step Three | 7mg |
|  | CLASSIC PATCH Step One | 21mg |
|  | Mint Gum | 2mg, 4mg |
|  | Lozenge Pre-Quit | 4mg |
|  | Lozenge - Original | 2mg, 4mg |
|  | Lozenge - Mint | 2mg, 4mg |
|  | Mini Lozenges - Mint | 1.5mg, 4mg |
|  | Mini Lozenges - Orange | 1.5mg |
|  | Strips - Mint | 2.5mg |

## Data management plan

## Description of the data

Types of data:

- Study administration data (i.e., Participant name, address, contact details, appointment dates)
- Patient clinical data (i.e., Data generated from baseline and outcome measures (i.e., PHQ-9, smoking history),
- Interview data

## Format and scale of the data

The study administration data will be stored as usual on IAPTUS or PCMIS, which are IAPTs client data management databases. To maintain workflow of the project Clinical Studies Officer/Local Trust Researchers store participants’ administration data on an Excel spreadsheet on NHS PCs, only on NHS premises.

The clinical data are recorded on IAPTUS or PCMIS, then input into the Bristol Medical School’s RedCap System. We anticipate recruitment of 158 participants with approximately 100 variables per participant. This volume of data is easily handled in RedCap.

Interview data will be stored in .doc format for immediate use, and in .pdf for long-term use, and audio files will be stored in an .mp3 format on the University of Bristol’s Research Data Storage Facility (<https://data.bris.ac.uk/data/>). We anticipate recruitment of approximately 40 participants for interviews. This volume of data is easily handled in the Research Data Storage Facility.

## Methodologies for data collection/ generation

See Figure 2 the data flow diagram outlining the methods for data collection, management, storage and sharing and the lifecycle of the data. All file-level encryption will be processed using 7-Zip software ([www.7-zip.org](http://www.7-zip.org)) on encrypted laptops/personal computers, and audio data will be encrypted-password protected by using the ‘Protect Audio Recorder with Encryption’ (<https://launchkit.io/websites/rgh8Z9vInzo/>) application on an encrypted Apple iPad (<https://www.apple.com/business/docs/iOS_Security_Guide.pdf>).

Figure 2 Flowchart of data methods and lifecycle

| Identifying potentially eligible participants |
| --- |
| Potentially eligible patients will be identified by IAPT database administrator/Clinical Studies Officers/Local Trust Researchers using the IAPTUS or PCMIS system; these data will remain on the NHS systems and on site at the Avon and Wiltshire Mental Health Partnership Trust/Oxford Health Trust. The data will be accessed and stored by the NHS IAPTUS or PCMIS System. This data will not be stored on any university systems or computers. |
|  |
| Data collection and transfer |
| *Study administration data:* Participant names, contact details, GP contact details, and interview dates will be collected via IAPTUS or PCMIS. These data will only be accessible on NHS premises and collected as part of usual IAPT care, and will be accessible by those involved in the study, and any other NHS staff involved in the clients’ care. Patient’s IAPTUS/PCMIS number will be used as a patient identifier and input into RedCap system held on the MySQL server during conduct of the trial.  *Patient clinical data:* These data will be collected by Psychological Wellbeing Practitioners and Clinical Studies Officers/Local Trust Researchers. Data will be input onto IAPTUS or PCMIS, and then extracted and input by Clinical Studies Officers/Local Trust Researchers on to the Bristol Medical School’s RedCap system during the conduct of the trial. The RedCap system is a secure database used for collecting, organizing and working with clinical trial data. The system is an online accessible platform, with a secured offline facility for collecting data when no internet access is available, data is uploaded once internet connection is recovered. Where it is not possible to collect data using RedCap, the data will be recorded via paper Client Report Forms (see appendix), and data will be input by Clinical Studies Officers/Local Trust Researchers onto the RedCap system at a later date. Paper forms will be kept on Trust premises in a locked filing cabinet in a locked office, until input on the RedCap, once input onto RedCap the paper forms will be destroyed at the project end date.  *Interview data:* These data will be collected using an encrypted password protected Apple iPad application, and stored in .mp3 format. During interviews neither IAPT clients, nor staff, nor any other person will purposefully be named - alternative names/IDs will be used. These data will then be uploaded to the Research Data Storage Facility (RDSF) and deleted from the Apple iPad. These data will be transferred to Bristol Transcription Services via their secured website for transcription (http://www.bristoltranscriptionservices.co.uk/). Any participant identifiers will be removed from the transcripts, and transcripts will be stored in .doc format on the Research Data Storage Facility. Audio data will be stored in encrypted password protected .mp3 files on the Research Data Storage Facility for the duration of the project, and will then be destroyed. |
|  |
| Working data storage |
| Study administration data will be used to maintain contact and workflow for the project will be: 1) collected and stored on IAPTUS or PCMIS as part of routine IAPT care, and will only be accessible by NHS employees as part of any routine care, and by those involved in the study. 2) Will also be input by Clinical Studies Officers/Local Trust Researchers into an excel spreadsheet in .xlsx format to maintain contact and workflow – this spreadsheet will be stored on NHS PCs, on Trust Premises only, and will only be accessed by those involved in the study.  Once the trial has ended the patient clinical data will be exported from Bristol Medical School’s RedCap system into .csv files and stored on the audited file server, here it will be stored for the duration of the project. The audited file server is a secured server used routinely for collection of NHS patient data for clinical trials purposes and meets the security requirements set forth by NHS Digital. The clinical data will be converted to Stata .dta format for cleaning and analysis.  Interview transcripts .doc, audio .mp3 data will be stored on the Research Data Storage Facility for the duration of the project. The Research Data Storage Facility is a secure system designed for storage of sensitive data. The audio and transcript data will be converted to N-Vivo format for analysis. |
|  |
| Data sharing |
| *Clinical and transcript data:* Upon completion of the project, anonymised data will be uploaded to the University of Bristol’s Research Data Repository (<https://data.bris.ac.uk/data/>). The Data Repository is a private online sharing facility accessible in the public domain. All data will be anonymised, and data access will be restricted (<https://data.bris.ac.uk/sensitive-research-data>). Data is made available to approved bona-fide researchers, after they have signed a data access agreement, the person will be granted access to the University of Bristol’s Data Repository (<https://data.bris.ac.uk>) by the Research Data Services (<https://data.blogs.ilrt.org/>). Data stored in the repository will have a Data Object identifier (DOI) and will be mentioned during all dissemination of the project.  Administration and audio data: Will not be uploaded for sharing due to the identifiable nature of these data, and will be destroyed at the end of the project (March 2020). |

## Data quality and standards

Those involved in collecting or handling study data will undergo training about how to collect, store, manage and transport the data to ensure that data is protected: Information Security Course ([www.bristol.ac.uk/infosec/](file:///C:\Users\gmjta\Dropbox\CRUK%20Fellowship\Co-design\Protcol\www.bristol.ac.uk\infosec\)), Mandatory Information Security Training ([www.bristol.ac.uk/infosec/training/](file:///C:\Users\gmjta\Dropbox\CRUK%20Fellowship\Co-design\Protcol\www.bristol.ac.uk\infosec\training\)), Research Governance Training ([www.bristol.ac.uk/social-community-medicine/shortcourse/scintroresgov.html](file:///C:\Users\gmjta\Dropbox\CRUK%20Fellowship\Co-design\Protcol\www.bristol.ac.uk\social-community-medicine\shortcourse\scintroresgov.html)), and NHS Good Clinical Practice Training ([www.crn.nihr.ac.uk/learning-development/good-clinical-practice/](http://www.crn.nihr.ac.uk/learning-development/good-clinical-practice/)).

To ensure consistency of data collection those involved in data collection will develop a standard operating procedure (SOP) guide for data collection entry, and pilot the data entry procedures during the feasibility study. To ensure that data is being entered correctly the applicant will do a 10% check of data entry, and discuss any discrepancies and solutions with the research nurse. Missing data will be stored as “.”.

To ensure quality of data transcription, interview data will be transcribed by Bristol Transcription Services as recommended by the University of Bristol for transcription of sensitive data for research purposes. Bristol Transcription services have been approved to process data subject to the Data Protection Act, and have entered into a formal "Personal Data Processing Agreement" (<http://www.bristol.ac.uk/secretary/data-protection/>), which falls under the remit of the University of Bristol’s Data Protection Act (<http://www.bristol.ac.uk/secretary/data-protection/>). To ensure quality of data transcription a researcher based at the University of Bristol will do a 50% check of audio data against the transcripts.

### Data preservation strategy and standards:

The final, cleaned and anonymised trial outcome dataset, and final anonymised transcript data will be stored in .xlsx and .pdf files, and will be available for 20 years on the University of Bristol’s online Research Data Repository (<https://data.bris.ac.uk/>). Data access will be restricted due to the potentially sensitive nature of the data and the possibility of re-identification. Data access methods will be published in an online open access peer-reviewed journal, the Open Science Framework, Clinicaltrials.gov and during presentations and other communications of the data. Audio data will not be uploaded to the Research Data Repository as it will be potentially identifiable.

## Data security and confidentiality of potentially disclosive information

### Formal information/data security standards:

The Research Data Storage Facility (RDSF) is working toward the ISO standard. Policy for the use of the RDSF is available at: [www.acrc.bris.ac.uk/acrc/RDSF_policy_220814.pdf](file:///C:\Users\gmjta\AppData\Roaming\Microsoft\Word\www.acrc.bris.ac.uk\acrc\RDSF_policy_220814.pdf).

### RedCap security:

The clinical data are managed on the Bristol Medical School’s RedCap online system. Patient identifiable administration data are not stored on RedCap. RedCap uses MySQL to store data. RedCap uses a high availability MySQL Cluster that is managed University of Bristol Central IT Services and maintained in line with all their policies regarding security, resilience and backups (Information Security Policy - <https://www.bristol.ac.uk/infosec/policies/>). RedCap uses Table Based username and password security, and a granular security model so users only see the data and functionality they require. RedCap maintains an audit trail of all actions including data changes made to the system. Access to the system is via an encrypted SSL website. The Chief Investigator, Principal Investigators, Clinical Studies Officers/Local Trust Researchers, and Psychological Wellbeing Practitioners will have access to RedCap. After final follow-up, the data stored in RedCap will be converted to .csv file format and uploaded to the University of Bristol’s audited file server for data cleaning and analysis for the duration of the project.

### Main risks to data security:

This research will involve collecting personal and sensitive data about participant’s mental health state or personal lives, and sensitive data from Psychological Wellbeing Practitioners and Service Managers about the health care they provide and their employer. Human error is a risk to the data; thus, an adequate training programme will be set up. All persons involved in obtaining, recording, storing and analysing data will have attended a data protection and security courses. Information Security Course ([www.bristol.ac.uk/infosec/](file:///C:\Users\GT15943\AppData\Roaming\Microsoft\Word\www.bristol.ac.uk\infosec\)); Mandatory Information Security Training ([www.bristol.ac.uk/infosec/training](file:///C:\Users\gmjta\AppData\Roaming\Microsoft\Word\www.bristol.ac.uk\infosec\training)); Data Protection Act Training ([www.icre8.org.uk/org/Courses/uob/dpa](file:///C:\Users\gmjta\AppData\Roaming\Microsoft\Word\www.icre8.org.uk\org\Courses\uob\dpa)). The Chief and Principal Investigators will also attend Good Clinical Practice Training delivered by the NHS (<http://www.nihr.ac.uk/our-faculty/clinical-research-staff/learning-and-development/national-directory/good-clinical-practice/>), which discusses NHS data protection and IAPT client data.

Data will not be copied onto external or personal hard drives. Any identifying information in transcriptions will be removed. However, the risk of re-identification is possible, researchers involved in the study will be bound to confidentiality regulations set out by the University of Bristol ([http://www.bristol.ac.uk/secretary/data-protection](http://www.bristol.ac.uk/secretary/data-protection/)/) and the NHS (<https://www.england.nhs.uk/wp-content/uploads/2016/12/confidentiality-policy-v3-1.pdf>). Data sharing will be restricted, and will only be made available to approved bona fide researchers, after they have signed a data access agreement, the person will be granted access to the University of Bristol’s Data Repository (<https://data.bris.ac.uk>) by the Research Data Services (<https://data.blogs.ilrt.org/>). The University of Bristol has a policy concerning the sharing of potentially re-identifiable data (<https://data.bris.ac.uk/sensitive-research-data/>).

It is not possible to rule out the threat of loss or damage to data, in this case we will follow standard university procedures as outlined in the “Reporting Lost/Stolen Data/Hardware Procedure” ([www.bristol.ac.uk/infosec/uobdata/reportloss](http://www.bristol.ac.uk/infosec/uobdata/reportloss)). To ensure security in this type of instance, clinical data and interview data will be stored on separate servers, and administration data will remain only on NHS premises.

## Data sharing and access

### Suitability for sharing:

This project will have a restricted-access sharing policy because of the sensitive nature of the data being collected from participants (<https://data.bris.ac.uk/sensitive-research-data/>). Data will be made available to approved bona fide researchers, after they have signed a data access agreement, the person will be granted access to the University of Bristol’s Data Repository (<https://data.bris.ac.uk>) by the Research Data Services (<https://data.blogs.ilrt.org/>). Data will be stored for 25 years. Administration, transcript and audio data will be destroyed after the study period, and will not be shared.

### Discovery by potential users of the research data:

All data listed on the University’s online Research Data Repository ([https://data.bris.ac.uk](https://data.bris.ac.uk/)) will be noted formally in academic citations with a Digital Object Identifier (DOI). During dissemination to academic, NHS, and IAPT client collaborators – access to the Research Data Repository will also be noted. The study will have a webpage available on the University of Bristol’s website, which will also include a link to the online Research Data Repository and details about applying for access.

### Governance of access:

This will be a restricted-access dataset. Bona fide researchers will be able to apply for the data produced by this study via the University of Bristol’s Research Data Services (<https://data.blogs.ilrt.org/>).

### The study team’s exclusive use of the data:

Data from consenting participants will be uploaded to the online Research Data Repository (<https://data.bris.ac.uk>), once it has been anonymised and any personally indefinable information has been removed. Data will be uploaded within 6-months after the study ends.

### Restrictions or delays to sharing, with planned actions to limit such restrictions:

No data will be shared whereby IAPT clients have not consented to data sharing. Any data that potentially undermines IAPT client anonymity will have identifiable information (e.g., I work at The University of Bristol”, “my line manager is John Smith”) removed or anonymised, and if this is not possible the data will not be made available. Bona fide researchers will be able to access the data produced during this trial via applying to the University of Bristol’s Research Data Services (<https://data.blogs.ilrt.org/>). If the researcher is successful in gaining approval the data will be transferred securely via an encrypted method, with the password sent separately. Delays to data sharing may occur if the process of gaining approval takes longer than predicted, or if access is denied.

### Regulation of responsibilities of users:

The University of Bristol’s Research Data Repository data sharing agreement is available here: <https://data.bris.ac.uk/sharingdata/>.

## Informed consent form

To gain verbal consent, the participant **must verbally agree** to each of the following questions. Tick (✓) once client has agreed.

| ✓ | Consent criteria |
| --- | --- |
|  | Do you confirm that you have read the participant information sheet dated xx (xx)? You have had the opportunity to consider the information, ask questions and have had these answered satisfactorily. |
|  | Do you understand that your participation is voluntary and that you are free to withdraw at any time without giving any reason, without your medical care or legal rights being affected? |
|  | Do you understand that sections of my data collected during the study, may be looked at by individuals from the University of Bristol? Do you give permission for these individuals to have access to my data under conditions of confidentiality? |
|  | Do you understand that the data collected from you as part of the study will be anonymised, and will be made “restricted-access data”? |
|  | Do you understand that the information collected about you will be used to support other ethically approved research in the future, and may be shared anonymously with other researchers? |
|  | Do you understand that the University of Bristol may use the data collected for this study in a future ethically approved research project but that the conditions under which you have provided the data will still apply? |
|  | Do you agree to take part in the study? |
|  | Do you understand that you may be contact by one of our researchers after 3-month follow-up for an interview about your experience of the study? |
|  | Do you agree for the research team to write to your GP to notify them of your participation, and the treatment that you receive as part of the study? |
|  | Do you understand that you will only be provided with nicotine preplacement products (e.g. patches, inhaler, etc.) for 12 weeks from start of treatment? If you require nicotine products beyond that point you will be able to obtain a prescription from your GP. Champix will be prescription only, and there will be a dispensing fee unless you receive your prescriptions for free via the NHS. |
|  | Do you understand researchers may listen to, transcribe or sit in on your treatment sessions to check the quality of the treatment you’re receiving? |
|  | Do you agree to be randomised to treatment condition? |

Researcher signature to confirm that the participant orally consented to the above study conditions:

|  |
| --- |

**Figure 3 Decision tree for adverse event reporting (non-CTIMPS)**


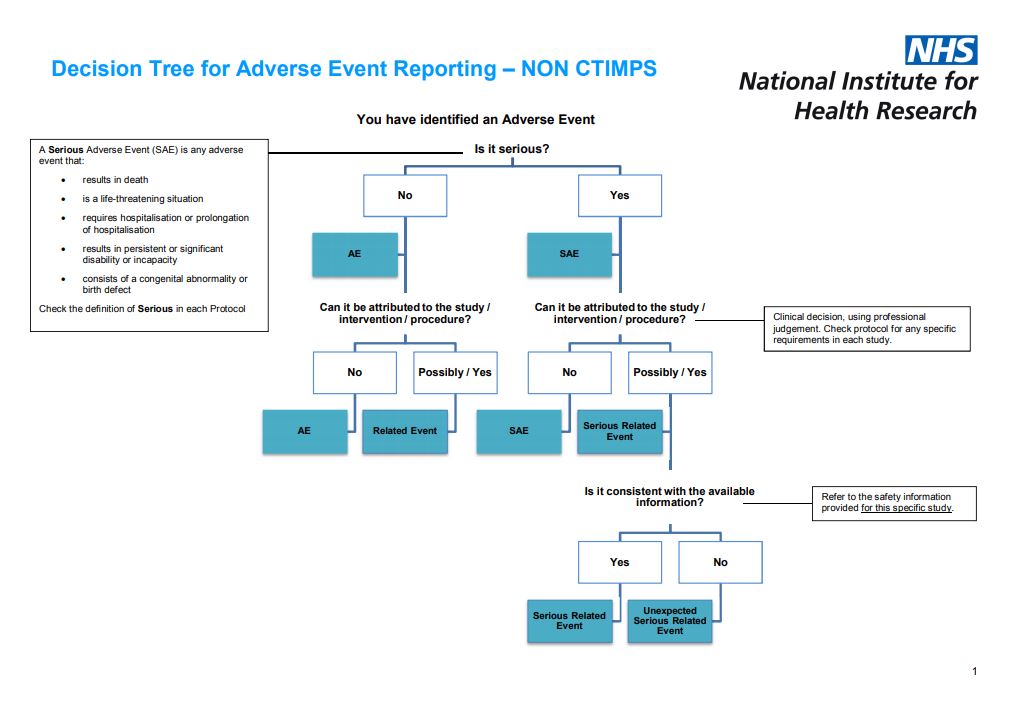

Supplement: Supplementary file 1 — Online appendix for the ESCAPE trial. (DOCX 125 kb) [file 40814_2018_385_MOESM1_ESM.docx]
